# Evaluation of ^18^F labeled glial fibrillary acidic protein binding nanobody and its brain shuttle peptide fusion proteins using a neuroinflammation rat model

**DOI:** 10.1371/journal.pone.0287047

**Published:** 2023-06-14

**Authors:** Takahiro Morito, Ryuichi Harada, Ren Iwata, Yoichi Ishikawa, Nobuyuki Okamura, Yukitsuka Kudo, Shozo Furumoto, Kazuhiko Yanai, Manabu Tashiro

**Affiliations:** 1 Division of Cyclotron Nuclear Medicine, Tohoku University Graduate School of Medicine, Sendai, Miyagi, Japan; 2 Department of Pharmacology, Tohoku University Graduate School of Medicine, Sendai, Miyagi, Japan; 3 Institute of Development, Aging and Cancer, Tohoku University, Sendai, Miyagi, Japan; 4 Division of Radiopharmaceutical Chemistry, Cyclotron and Radioisotope Center, Tohoku University, Sendai, Miyagi, Japan; 5 Division of Pharmacology, Faculty of Medicine, Tohoku Medical and Pharmaceutical University, Sendai, Miyagi, Japan; Babasaheb Bhimrao Ambedkar University (A Central University), INDIA

## Abstract

Astrogliosis is a crucial feature of neuroinflammation and is characterized by the significant upregulation of glial fibrillary acidic protein (GFAP) expression. Hence, visualizing GFAP in the living brain of patients with damaged central nervous system using positron emission tomography (PET) is of great importance, and it is expected to depict neuroinflammation more directly than existing neuroinflammation imaging markers. However, no PET radiotracers for GFAP are currently available. Therefore, neuroimaging with antibody-like affinity proteins could be a viable strategy for visualizing imaging targets that small molecules rarely recognize, such as GFAP, while we need to overcome the challenges of slow clearance and low brain permeability. The E9 nanobody, a small-affinity protein with high affinity and selectivity for GFAP, was utilized in this study. E9 was engineered by fusing a brain shuttle peptide that facilitates blood-brain barrier permeation via two different types of linker domains: E9-GS-ApoE (EGA) and E9-EAK-ApoE (EEA). E9, EGA and EEA were radiolabeled with fluorine-18 using cell-free protein radiosynthesis. *In vitro* autoradiography showed that all radiolabeled proteins exhibited a significant difference in neuroinflammation in the brain sections created from a rat model constructed by injecting lipopolysaccharide (LPS) into the unilateral striatum of wildtype rats, and an excess competitor displaced their binding. However, exploratory *in vivo* PET imaging and *ex vivo* biodistribution studies in the rat model failed to distinguish neuroinflammatory lesions within 3 h of ^18^F-EEA intravenous injection. This study contributes to a better understanding of the characteristics of small-affinity proteins fused with a brain shuttle peptide for further research into the use of protein molecules as PET tracers for imaging neuropathology.

## Introduction

Neuroinflammation is characterized by activated microglia and reactive astrocytes, which contributes to neurodegeneration in various neurological conditions, such as Alzheimer’s disease (AD) [1–4]. Notably, activated microglia and reactive astrocytes have been found in the vicinity of misfolded proteins, such as amyloid plaques and tau tangles in the brains of patients with AD, indicating a crucial relationship between neuroinflammation and AD pathogenesis [5]. Cross-sectional postmortem studies revealed that the number of glial fibrillary acidic protein (GFAP)-positive astrocytes and activated microglia were correlated with tau tangles; however, not with amyloid plaques in AD. Although knowledge on neuroinflammation has been rapidly accumulating in recent years, it is still unclear how reactive glial cells function in neurodegenerative conditions or whether they actively contribute to neurodegeneration in the living human brain [6,7]. *In vivo* imaging of glial cell status using positron emission tomography (PET) would provide new insights into understanding better the disease mechanism, accurate diagnosis, and clinical drug development [8].

The most prominent marker for reactive astrocytes is GFAP, an abundant type III intermediate filament protein and a potential imaging target of astrogliosis [9]. To date, no small molecules with high affinity and selectivity against GFAP have been reported, possibly due to its simple α-helix structure, which makes identifying the particular binding site of GFAP challenging. Therefore, PET radiotracers for alternative reactive astrogliosis markers have been proposed and tested in rodent and human models [8]. Translocator protein 18kDa (TSPO), a protein found in the outer membranes of both activated microglia and reactive astrocytes, has been the subject of numerous investigations in preclinical and clinical studies [10–18]. TSPO is widely recognized as an imaging marker for activated microglia because of its high expression in activated microglia [15,19]. The enzyme monoamine oxidase B (MAO-B), which is primarily expressed in the outer mitochondrial membrane of astrocytes, is upregulated in reactive astrocytes and is used as an astrogliosis marker [8,20–22]. MAO-B PET tracers show high tracer binding in vulnerable areas, which are expected to cause astrogliosis in humans [8,21,22]. Imidazoline_2_ binding sites (I_2_BS), defined as a group of heterogeneous proteins preferentially recognized by I_2_BS ligands, such as idazoxan, are also upregulated in reactive astrocytes [23]. In some neurodegenerative diseases, the I_2_BS PET tracer also exhibits elevated tracer retention in the human brain [24,25]. TSPO, MAO-B, and I_2_BS expression levels correlate with GFAP; however, they also have a non-negligible expression in cells other than astrocytes. In addition, a comparison study of TSPO and MAO-B PET tracers in a rodent model revealed that they are not always upregulated simultaneously, implying that multiple comparisons of neuroinflammation markers are important for a better understanding of neuroinflammatory events *in vivo* [18]. GFAP, in particular, has the potential to be a direct astrogliosis PET marker due to its intense and relatively specific expression in reactive astrocytes.

Target-specific high-affinity proteins have been developed and applied for molecular imaging and radioimmunotherapy to date, including antibodies, nanobodies, and affibodies [26]. We previously reported that the fusion of a brain shuttle peptide ApoE(159–167)_2_, which reportedly induces receptor-mediated permeation of its cargo protein into the brain by binding to low-density lipoprotein receptor-related protein 1 (LRP1), increased the brain uptake of AS69, an affibody-based α-synuclein binding protein (14 kDa) [27–29]. Moreover, clearance of the fusion protein from the brain was initiated 120 min after intravenous injection in mice [29]. These results suggest that pathological alterations in the brain can be visualized *in vivo* using PET and ^18^F-labeled small-affinity proteins.

In the present study, a nanobody E9, which is also a small affinity protein (14 kDa) that binds GFAP with low-nanomolar affinity (reportedly *K*_D_ = 5.6 nM determined by enzyme-linked immunosorbent assay), was chosen as a protein radiotracer candidate for GFAP *in vivo* imaging [30–32]. Then, E9 was engineered for brain delivery by fusing ApoE(159–167)_2_ with two different types of linkers: the flexible and rigid linkers, to compare the effect of the linker on protein expression, binding affinity, specificity to GFAP, and radiosynthesis efficiency [33]. Finally, we evaluated the protein radiotracers in a rat model injected with lipopolysaccharide (LPS) into the unilateral striatum, which is generally used in PET tracers to assess neuroinflammation [34–36]. Investigations for *in vivo* GFAP imaging in this study will highlight the challenges of using proteins as PET agents and help further development of protein PET tracers.

## Materials and methods

### Gene design, bacterial expression, and purification of E9, EGA, and EEA nanobodies

**Fig 1:**
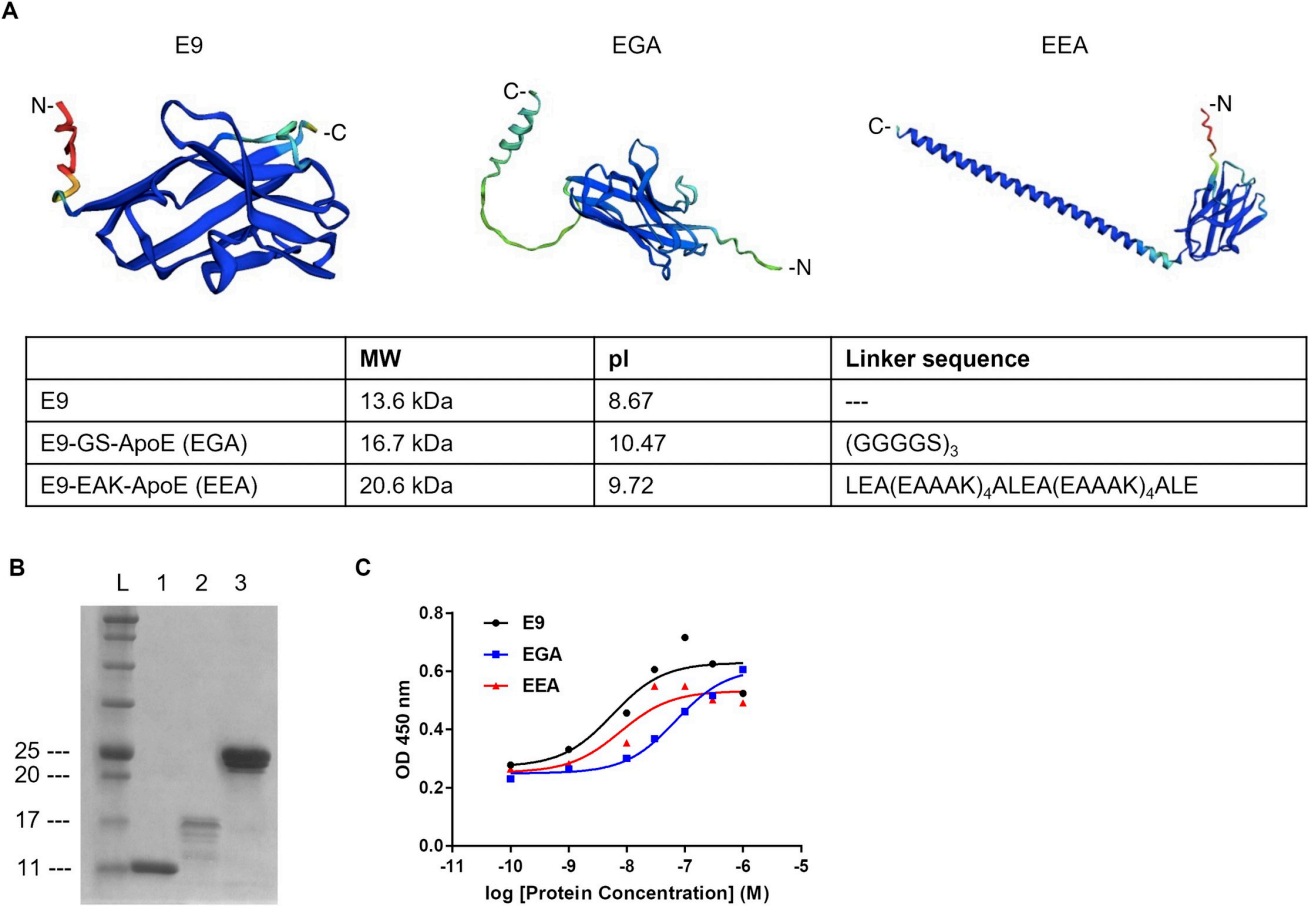
Characterization of GFAP-specific binding protein E9 and designed molecules E9-GS-ApoE (EGA) and E9-EAK-ApoE (EEA). (A) Predicted structures and characteristics of E9, EGA and EEA. Ribbons were colored with predicted local distance difference test score per position calculated at the same time. MW: Molecular weight, pI: Isoelectric point, N: N-terminal, C: C-terminal. (B) SDS-PAGE analysis of purified E9, EGA and EEA. L: ladder, 1: E9, 2: EGA, 3: EEA. (C) ELISA analysis for comparison of E9, EGA and EEA affinity to GFAP from rat brain homogenates. OD: Optical density.

The amino acid sequence of E9 was described by Li *et al*. [32]. Using this sequence, we designed two types of E9 derivatives (EGA, EEA) using the sequence of flexible linker <(GGGGS)_3_>, rigid linker <LEA(EAAAK)_4_ALEA(EAAAK)_4_ALE>, and ApoE (159–167)_2_ peptide <(LRKLRKRLL)_2_>. The sequences were custom-synthesized by Eurofin Genomics (Tokyo, Japan) and subcloned into the pET-21a or pET-28a plasmid vectors with the restriction site *Nde*I/*Xho*I for bacterial expression. The His_6_-tag was fused at the C-terminus (E9) or N-terminus (EGA and EEA) of the genes for protein purification and immunodetection. ColabFold was used to predict the protein structures (Fig 1A) [37]. Plasmids were used to transform SHuffle T7 Express Competent E. coli for bacterial expression (New England Biolab Japan Inc., Tokyo, Japan). After overnight incubation in 5 ml Luria-Bertani medium with appropriate antibiotics, 1 ml of the bacterial solution was added to 200 ml medium and incubated for 3–4 h until the optical density at 500 nm reached 0.5–0.7. The cells were then collected by centrifugation (2580 × g, 30 min, 4°C) and stored at -80°C for the next purification step. Protein purification was performed using two kinds of chromatography. First, the cells were lysed by sonication on ice in phosphate-buffered saline (PBS) containing 20 mM imidazole, 0.1 U/ml benzonase (Merck, Rahway, NJ), 0.1% Triton X-100, and 1 tablet/50 ml cOmplete^TM^ Proteinase Inhibitor Cocktail (Roche, Basel, Switzerland). The muddy solution was centrifuged (20,000 × g, 30 min, 4°C), and the supernatant was collected and filtered through a 0.22 μm diameter filter (SLGVV255F, Merck). The flow-through was diluted 2- to 5-fold in PBS containing 20 mM imidazole before loading it onto a HisTrap FF column (1 ml, Cytiva, Marlborough, MA) for immobilized metal affinity chromatography using the AKTA start system (Cytiva). After 10-column volume (CV) washing with PBS containing 20 mM imidazole, the product was eluted using PBS containing 500 mM imidazole. Following a 10-fold dilution with 50 mM phosphate buffer (pH 7.2), the eluent was loaded onto a HiTrap SP HP column (1 ml, Cytiva) for cation-exchange chromatography. After 10 CV washes with 50 mM phosphate buffer (pH 7.2), the retained proteins were eluted using a 10 CV gradient with 50 mM phosphate buffer (pH 7.2) and 1M NaCl. Typically, E9 elution occurred earlier than EGA and EEA elution, as expected from the difference in their isoelectric points. Product elution was confirmed by sodium dodecyl sulfate-polyacrylamide gel electrophoresis (SDS-PAGE). The elution fractions were diluted with PBS more than 50-fold and ultracentrifuged to less than 1 ml using an Amicon Ultra-15 ml, 3000 molecular weight cut-off (Merck). Protein concentration was determined using a Pierce^TM^ BCA Protein Assay Kit (Thermo Fisher Scientific, USA).

### Animals and surgeries

The Laboratory Animal Care Committee of Tohoku University approved all animal protocols. All animal experiments were performed per the relevant guidelines and regulations, including the ARRIVE guidelines (https://arriveguidelines.org/). All animals were generally housed in cages on a 12 h light/12 h dark cycle at 21–23°C and 50–80% humidity, with free access to water and food. E. coli O55:B5-derived LPS (L2880, Merck) was injected into the unilateral striatum of rats (slc: Wistar, male, 13–15 w) to create a rat model of unilateral neuroinflammation. First, the rats were anesthetized with 3.0% isoflurane by inhalation until unconsciousness. Next, a mixture of three anesthetics (medetomidine hydrochloride 0.75 mg, midazolam (10 mg), and butorphanol tartrate (12.5 mg) in 50 ml PBS) was intraperitoneally injected with 0.05–0.08 ml/kg. Following sedation, the hair was cut, the head was fixed with a stereotactic fixing tool, the skull was exposed, and a hole for microinjection was drilled. Then, using a 10 μl Hamilton syringe and a motorized stereotaxic microinjector IMS-20 (NARISHIGE, Tokyo, Japan), 4 μl of LPS (5 μg/μl concentration) was injected at a rate of 0.5 μl/min into the designated coordinate (A.P.: + 0.5 mm, ML: left 3 mm and DV: - 4.3 mm [34]). The needle was slowly retracted 2 min after the injection, and the hole was filled with PBS-wetted cotton and dental cement. Finally, the scalp was sutured with threads, and the rats were returned to the home cages.

### Immunohistochemistry

The rats were sedated with 3.0% isoflurane and the anesthetics mixture (0.1 ml/kg) mentioned above a week after the LPS injection. The rats were then perfused with 100 ml saline, followed by 100 ml of 10% formalin-neutral buffer solution (Fujifilm-Wako). The brains were collected and kept at 4°C in a 10% formalin-neutral buffer solution. The buffer was replaced with 30% sucrose the day before cutting with a cryostat for cryoprotection (CM3050S; Leica, Wetzlar, Germany). Brain coronal sections with a 40-μm thickness were created and stored in PBS containing 0.02% sodium azide for up three months. The brain sections were blocked with 3% horse serum in PBS containing 0.2%Tween20 (PBST) before incubation overnight with 5–10 μg/ml purified E9, EGA, or EEA nanobodies. Following three 2 min washes with PBST, the sections were incubated with primary antibodies in blocking buffer (mouse anti-His-tag antibody [1B7G5, Proteintech, Japan]:1:1000, rabbit anti-GFAP antibody [422261, Nichirei Bioscience, Tokyo, Japan]:1:2, rabbit anti-LRP1 antibody EPR3724 [ab92544, Abcam, Cambridge, UK]:1:300, and rabbit anti-Iba1 antibody ([019–19741, Fujifilm-wako]:1:1000). If LRP1 staining was performed, the brain sections were incubated for 10 min at 95°C in antigen unmasking solution (pH 9.0, H-3301, Vector Laboratories) before incubation of the nanobody. They were washed three times for 5 min each before incubation with secondary antibodies in blocking buffer (goat anti-mouse IgG H&L conjugated with Alexa Fluor 647 [A32728, Invitrogen]:1:500, goat anti-rabbit IgG H&L conjugated with Alexa Fluor 488 [A11008, Invitrogen]:1:500). After three times wash with PBST for 5 min, the sections were mounted with FluorSave (Merck) and observed under a fluorescence microscope (Eclipse 80i; Nikon, Tokyo, Japan).

### Semi-quantification of GFAP by Western blot

Rats were perfused with saline for 1 week after injection, and the brain was extracted. The brain was first cut in the coronal direction at the point of lambda and + 3.7 mm from the bregma in the coronal direction to remove the olfactory bulb and cerebellum, and then in the sagittal direction at the midline to separate left brain (LPS-injected side) and right brain (contralateral side). Each brain homogenate was prepared in PBS (0.1 g tissue/mL) and preserved at -80°C. The protein concentration of brain homogenates was determined by BCA assay and adjusted to 1 mg/ml for western blot analysis. The sample fractions were mixed with an equivalent Sample Buffer Solution (Fujifilm-Wako) and boiled at 95°C for 3 min. Following SDS-PAGE, proteins in the gel were transferred to the LF-PVDF membrane (Bio-Rad, Hercules, CA, USA) previously soaked in methanol for 3 min and Trans-Blot Turbo Transfer buffer (Bio-Rad) for 3 min, as described in the manual. After 5 min or overnight incubation in EveryBlot Blocking Buffer (Bio-Rad), the membrane was incubated in primary antibody solution (GA5 [Merck] 1:4000, diluted with 4 ml EveryBlot Blocking Buffer) for 1 h at 20–25°C. Next, the membrane was washed five times with Tris-buffered saline and 0.05% Tween20 (TBST) for 5 min, followed by incubation in a secondary antibody solution (StarBright^TM^ Blue 700 goat anti-mouse IgG antibody [Bio-Rad] 1:2500, hFAB^TM^ Rhodamine anti-actin antibody [Bio-Rad] 1:2000) for 1 h at 20–25°C. The membrane was washed six times with TBST for 5 min, and fluorescence was observed using a ChemiDoc Touch MP (Bio-Rad). Image analysis was performed using the ImageLab software (Bio-Rad).

### Enzyme-linked immunosorbent assay (ELISA)

PBS containing 0.1% Triton X-100 (8 mg/ml protein concentration) was used to homogenize rat brains. The GFAP homogenates were centrifuged at 20,000 × g for 30 min, and the supernatant was diluted to 100 μg/ml in PBS containing 0.1% bovine serum albumin (BSA) (PBSB). Then, various concentrations of E9, EGA, and EEA diluted in PBSB were mixed with GFAP extract (1:1) and incubated for 1 h at 20–25°C. The mixture (200 μL) was transferred to Ni-NTA HisSorb Plates (QIAGEN, 35061, USA) to capture His-tag-fused E9, EGA, and EEA at the bottom of the plate. Captured GFAP was detected using a rat anti-GFAP monoclonal antibody (Invitrogen, 2.2B10, 1:5000) after four washes with PBST. Following the same washing procedure, horseradish peroxidase-conjugated anti-rat IgG (Proteintech, 1:5000) was added to the plate for 1 h at 20–25°C. Following washing, ELISA TMB Substrate (ab171523, Abcam) was added to the plate for 2–5 min, and the reaction was stopped with 450 nm Stop Solution for TMB Substrate (ab171529, Abcam). The absorbance at 450 nm was measured using a plate reader SpectraMAX M2 (Molecular Devices, USA). The assay was performed in duplicates. The values of half-maximal effective concentration (EC_50_) were calculated using GraphPad Prism 7.

### Preparation of additional components for cell-free protein radiosynthesis

As previously reported, our cell-free protein radiosynthesis (CFPRS) system was based on a cell-free translation system [23,32]. CFPRS requires additional components, such as plasmid DNA, the engineered aminoacyl-tRNA synthetase (*p*CNF-RS), its tRNA (tRNA_CUA_^opt^) pair, and a radiolabeled amino acid (*O*-(2-[^18^F]fluoroethyl)-L-tyrosine ([^18^F]FET)). Cell-free translation system reagent (RTS 100 E. coli HY Kit) was obtained from Biotech Rabbit GmbH (Berlin, Germany). Engineered tRNA (tRNA_CUA_^opt^) was custom-synthesized by AJINOMOTO BIO PHARMA SERVICES (Tokyo, Japan). Using site-directed mutagenesis PCR, the amber codon TAG was inserted next to the start codon ATG of the template plasmid DNAs encoding E9, EGA, or EEA. The product plasmids were used for transformation with Champion^TM^ DH5α high (SMO Bio, Japan) after their sequence was confirmed. The final plasmid DNA solution (>300 ng/μL) was prepared using the NucleoBond Xtra Maxi Plus (TAKARA BIO Inc., Japan). The gene of *p*CNF-RS was custom-synthesized (Genscript, USA) and subcloned into the pET-52b plasmid to fuse the strep-II affinity tag [38]. The plasmids were transformed into BL21 (DE3) cells, and the proteins were expressed and extracted as described above. PBS was used as a binding buffer, and PBS containing 2.5 mM d-desthiobiotin (D1411, Sigma-Aldrich, St. Louis, MO) as an elution buffer for affinity chromatography using StrepTrap HP column (Cytiva). Using single-affinity chromatography, the *p*CNF-RS product was obtained with > 95% purity in SDS-PAGE gel stained with Coomassie brilliant blue (S1 Fig). The elution fraction solvent was replaced with PBS using a PD-10 column (Cytiva), and the eluent was concentrated to 8 mg/ml by ultracentrifugation, as described above. Fluorine-18 represents a radiological hazard and must be used only with institutional, state, and/or federal authorization and according to ALARA (as low as reasonably achievable) principles. Radiation from radionuclides was properly shielded by performing all reactions in a lead-shielded fume hood or hot cell in accordance with radiation safety guidelines. [^18^F]FET was synthesized as previously described [39]. After purification, the [^18^F]FET product diluted with 50 mM HCl aqueous solution was loaded into a tC18short cartridge (Waters, Milford, MA, USA) and eluted with ethanol. After evaporating, ethanol/water by azeotropic drying concentrated [^18^F]FET was prepared in Reconstitution Buffer (provided in the RTS 100 E. coli HY Kit). The molar activity was 211 ± 39 GBq/μmol immediately before protein radiosynthesis (n = 9).

### ^18^F labeling of proteins by cell-free protein radiosynthesis (CFPRS)

Radiolabeled proteins were prepared using cell-free translation as described elsewhere [29]. Briefly, the reaction mixture contained cell-free protein synthesis reagents (*E*.coli Lysate, Reaction Mix, Amino Acids, Methionine, provided by RTS 100 E. coli HY Kit), *p*CNF-RS (300 μg), tRNA_CUA_^OPT^ (50.3 μg), RNase inhibitor (300 U), template pET-21a plasmids (4.5 μg), and [^18^F]FET (201 ± 47 MBq) and incubated at 30°C for 30 min. The synthesized proteins were purified from the crude solution using a HisSpinTrap column (Cytiva) and NAP-5 column (Cytiva) according to the instructions of the manufacturer. Radiolabeled protein production was analyzed by gel autoradiography using NuPAGE^TM^ gel (12% Bis-Tris gel with MES-SDS Running buffer; Invitrogen). The gels were then exposed to an imaging plate (BAS-IP TR 2025, Cytiva) overnight. Autoradiographic images were acquired using a Typhoon FLA 9500 Laser Scanner (Cytiva). The molar activity of the purified proteins was estimated from that of [^18^F]FET.

### *In vitro* autoradiography

Brain floating sections were fixed on MAS-coated glass slides (Matsunami Glass Ind., Ltd., Osaka, Japan), dried for minutes, and soaked in assay buffer (PBS containing 0.5% BSA) for 30 min, and the solution was discarded immediately. Radiolabeled products were diluted with the assay buffer to a concentration of 370 kBq/ml, and the sections were incubated in the radioactive solution (100 μL/section) for 30 min. The protein concentrations were calculated as 5.49 nM (^18^F-E9), 2.38 nM (^18^F-EGA), and 6.94 nM (^18^F-EEA) from the molar activity of the proteins estimated from that of [^18^F]FET. The assay was performed in the presence of 5 μM unlabeled E9, EGA, and EEA to determine non-specific binding to tissues. Then, the sections were washed twice with the assay buffer for 5 min, PBS for 5 min, and dried for several minutes. The glass slides were then exposed overnight to an imaging plate. Autoradiographic images were acquired as previously described. Quantitative analysis was performed using ImageQuant^TM^ TL ver.8 (Cytiva).

### Small-animal PET imaging

A PET study was performed using a Clair vivo PET scanner (Shimadzu, Japan). Before the PET scans, rats (slc: Wistar, male, 14 w, n = 2) were anesthetized with 2.5% (v/v) isoflurane. Following intravenous administration of ^18^F-EEA (3.45 MBq [Rat 1] and 2.99 MBq [Rat 2] in 200 μl) dissolved in PBS via tail vein catheters, emission scans were acquired in three-dimensional list mode. The resulting sinograms were reconstituted using the three-dimensional DRAMA algorithm into several frames (Rat 1:1 min × 5, 2 min × 5, 5 min × 3, Rat 2:10 min × 6). In addition, standardized uptake value (SUV) images were obtained using AMIDE software by normalizing the tissue radioactivity concentrations based on the injected dose and body weight [40].

### *Ex vivo* biodistribution study

Neuroinflammatory model rats (five days after LPS injection, 12w, male, n = 3) were anesthetized, cervically dislocated, and dissected 3 h after intravenous injection of ^18^F-EEA (1.48 MBq/0.2 mL) via the tail vein. The tissues (left brain, right brain, cerebellum, liver, kidney, bone, heart, lung, spleen, stomach, small intestine, large intestine, bladder, muscle, and urine) were collected into vials, and radioactivity and weight were measured with a gamma counter AccFLEX γ7000 (Hitachi, Tokyo, Japan). To calculate the injected dose per gram (%ID/g), the tissue radioactivity was divided by the radioactivity of the injected radiotracer and the tissue weight, then multiplied by 100. The results are shown as the mean ± standard error of the mean (SEM) calculated using GraphPad Prism 7.

## Results

### Characterization of E9 and its derivatives

We investigated two types of linkers that “flexibly” or “rigidly” separate two domains of protein used in the fusion of two functional proteins [27]. First, we designed two proteins using each linker, E9-GS-ApoE (EGA) and E9-EAK-ApoE (EEA), with each linker for brain delivery of the E9 functional domain (Fig 1A). Their expression in *E*. coli and purity were confirmed by SDS-PAGE (Fig 1B). They were produced with the typical yield of 6.0 mg/1 L of medium for E9, 1.0 mg/1 L of medium for EGA, and 2.5 mg/1 L of medium for EEA after two purification steps of immobilized metal affinity chromatography and cation-exchange chromatography. To compare the binding ability of EGA and EEA with E9, we performed ELISA analysis by detecting GFAP bound to each affinity molecule at various concentrations in co-incubation with rat brain homogenates (Fig 1C). Their EC_50_ was calculated as 5.69 nM for E9 (95%CI:0.70–25.3 nM), 69.8 nM for EGA (95%CI:31.2–165 nM), and 7.80 nM for EEA (95%CI:1.61–25.6 nM). The affinity of EEA for GFAP was comparable to that of E9, whereas that of EGA was relatively low.

### E9, EGA, and EEA stained rat GFAP in a rat neuroinflammation model

**Fig 2:**
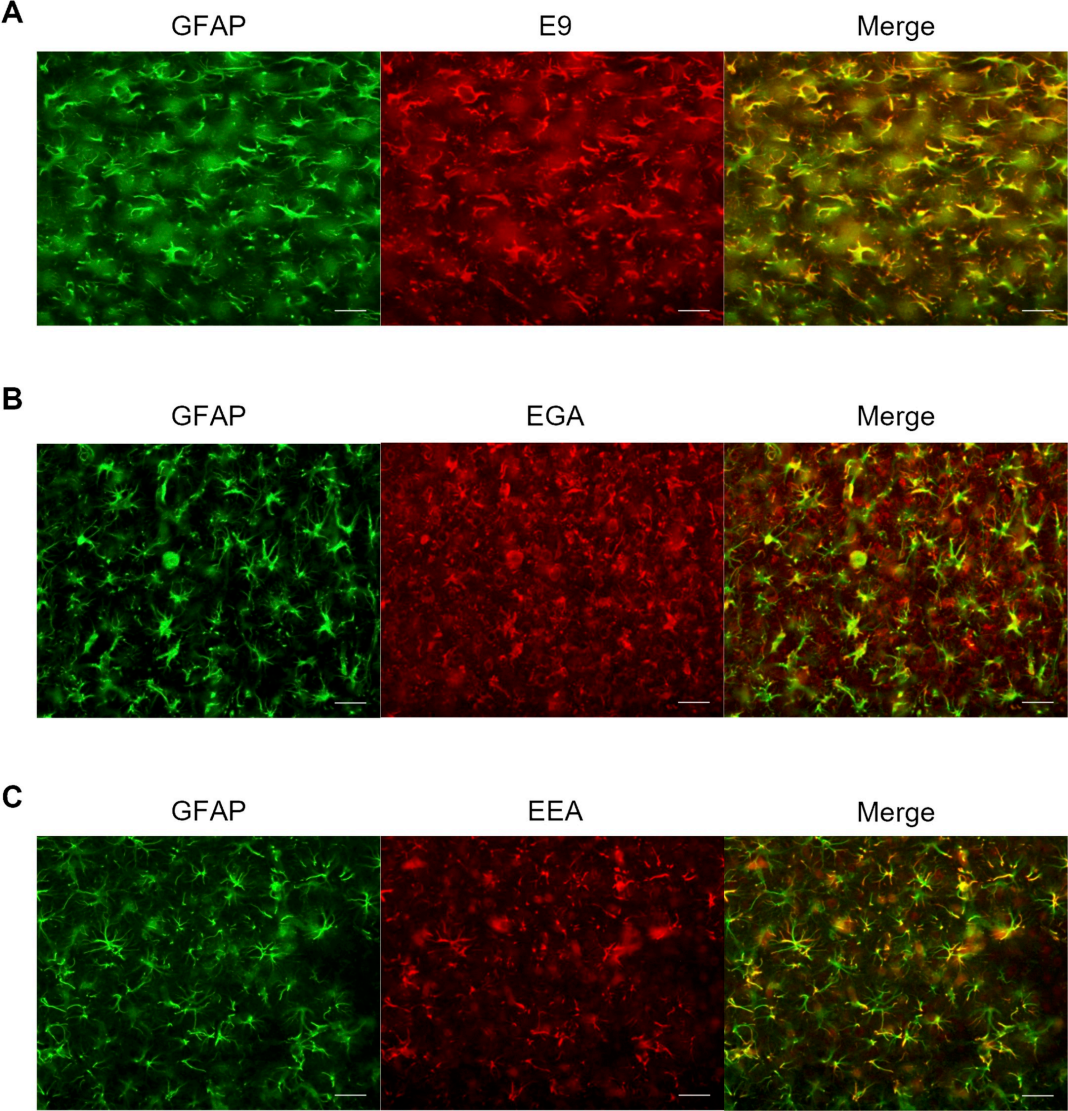
Immunohistochemical staining of rat brain sections (cortex) with general GFAP antibody GA5 and three affinity proteins. (A) E9, (B) EGA, and (C) EEA were used for immunostaining and compared with GA5-positive GFAP stains. White arrows indicate the representative position of EGA binding that is not GFAP. Scale bars: 100 μm.

We performed immunostaining on rat brain slices with E9, EGA, and EEA to confirm that ApoE(159–167)_2_ fusion to E9 does not affect E9 binding ability in a rat model of neuroinflammation that was stereotaxically injected with LPS into the left brain striatum as described in the literature [34–36]. This model showed upregulation of GFAP expression on the left side, as assessed using western blotting (S2B and S2C Fig). In addition, the neuroinflammation markers GFAP and Iba1 discriminated the LPS-injected brain side-specific significant increase in the neuroinflammatory response (S2D Fig), which is consistent with the results of previous studies [34–36]. Double immunofluorescence staining of the brain sections showed that E9, EGA, and EEA colocalized with GA5-positive astrocytes (Fig 2A–2C). However, EGA also stained GA5-negative circular structures. To confirm the binding target of EGA, we performed double immunostaining for LRP1, which is a reported ApoE(159–167)_2_ target molecule. Images obtained from the LRP1 antibody and the EGA nanobody were only partially merged (S3 Fig), implying that the non-GFAP binding of the EGA does not reflect the localization of LRP1.

### Radiosynthesis of ^18^F-E9, ^18^F-EGA and ^18^F-EEA by CFPRS

**Fig 3:**
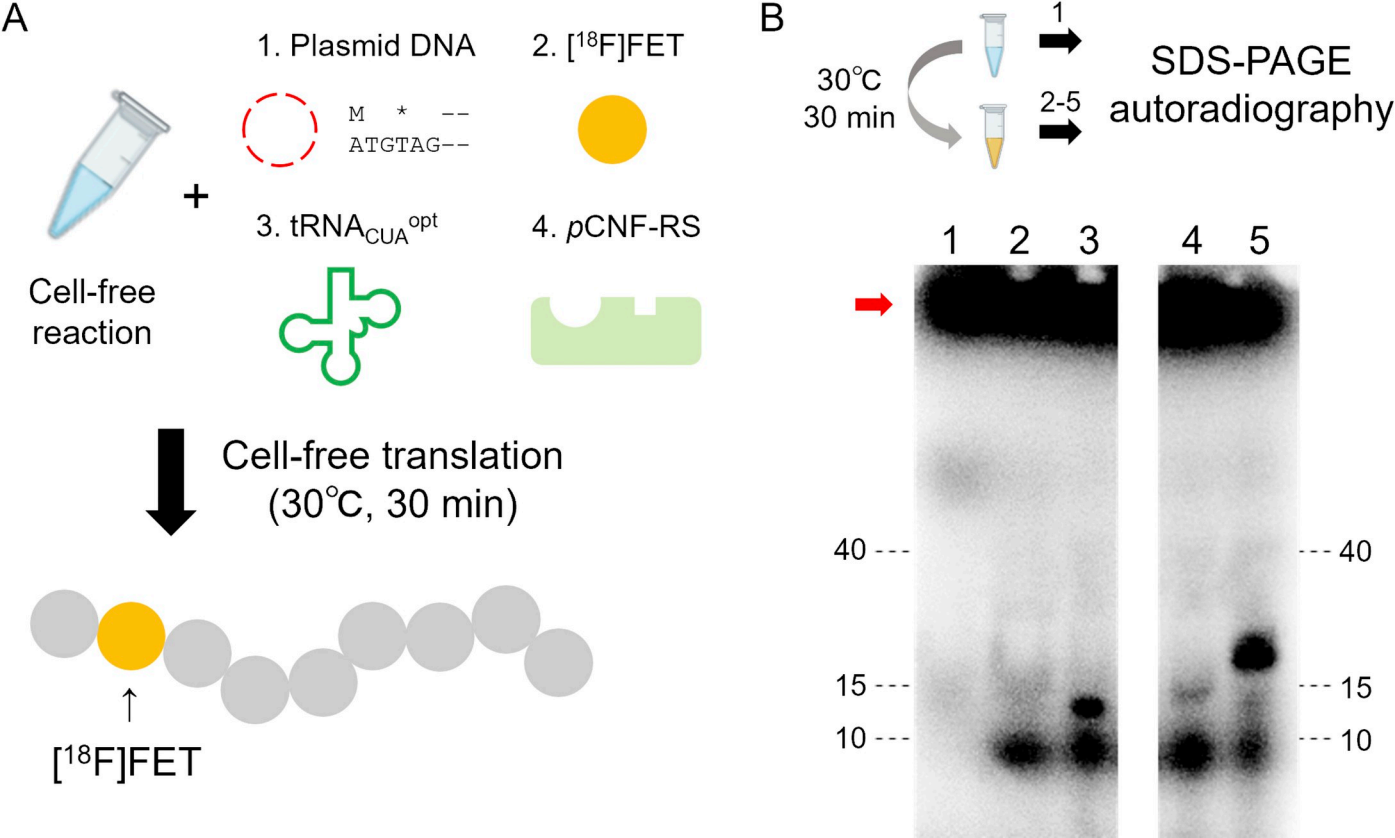
Cell-free protein radiosynthesis of protein tracers. (A) Brief description of ^18^F protein labeling method [41]. (B) Gel autoradiography to confirm synthesis of ^18^F-labeled proteins. A red arrow indicate the position of [^18^F]FET that typically remains at the top of the NuPAGE gel during this electrophoresis. 1: Before synthesis, 2–6: After synthesis [2: no DNA, 3: ^18^F-E9, 4: ^18^F-EGA, 5: ^18^F-EEA].

We prepared ^18^F-E9, ^18^F-EGA, and ^18^F-EEA using CFPRS which we developed as previously described [29,41]. Briefly, cell-free translation reagents were supplemented with four factors: plasmid DNA that encodes amber codon TAG after start codon ATG, [^18^F]FET, tRNA_CUA_^opt^, and *p*CNF-RS to engineer the genetic code by assigning an amber codon to [^18^F]FET, resulting in the incorporation of [^18^F]FET into the protein (Fig 3A). The production of these radiolabeled proteins was confirmed using gel autoradiography (Fig 3B). The radiochemical conversion of ^18^F-EEA was the highest, followed by ^18^F-E9 and ^18^F-EGA. Calculated from the intensity of the product bands, the ratio of ^18^F-EGA and ^18^F-EEA compared to ^18^F-E9 was determined as 0.74 and 1.5, respectively. The radiochemical yields of ^18^F-E9, ^18^F-EGA, and ^18^F-EEA were 4.61% (n = 6), 5.19% (n = 2), and 8.01% (n = 3), respectively.

### *In vitro* autoradiography in rat brain sections of neuroinflammation model

**Fig 4:**
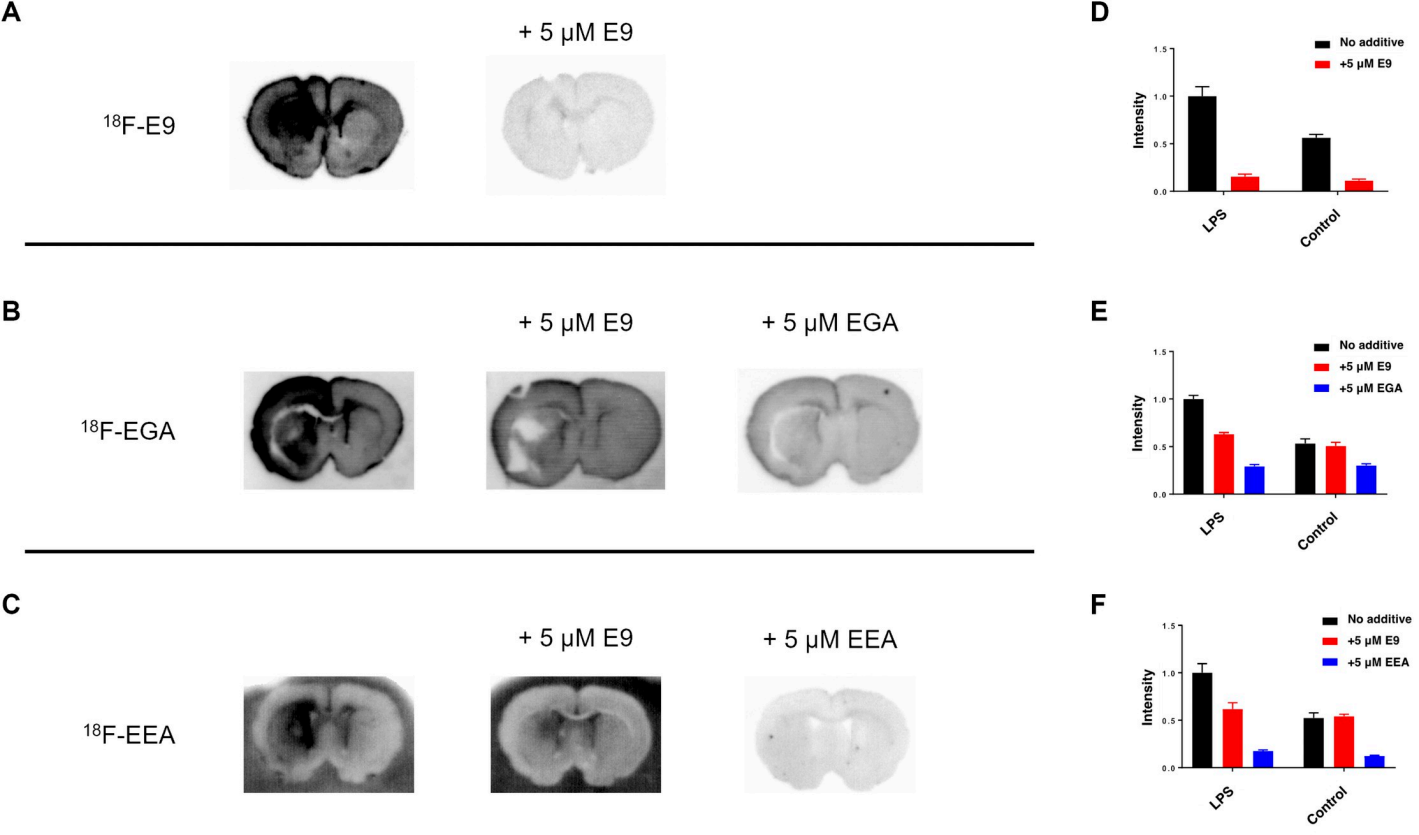
*In Vitro* Autoradiography in Rat Brain Sections of Neuroinflammation Model. (A-C) Autoradiographic images of ^18^F-E9 (A), ^18^F-EGA (B) and ^18^F-EEA (C) in rat brain sections of LPS-induced neuroinflammation model. Images of each row are displayed with the same contrast and obtained from the same experiment using the same lot rat brain sections and protein tracers (D-F). Quantitative analysis of images from A (D), B (E), and C (F).

*In vitro* autoradiography of ^18^F-E9 showed increased binding in the left ipsilateral striatum in LPS-induced inflammation rat brain sections. The binding was displaced by excess non-radiolabeled E9, indicating that specific binding of ^18^F-E9 to GFAP was detected in the brain sections (Fig 4A). Moreover, ^18^F-EGA and ^18^F-EEA showed increased binding in the left ipsilateral striatum in the brain section. Compared with ^18^F-E9, the binding of ^18^F-EGA and ^18^F-EEA remained detectable in the presence of excess non-radiolabeled E9; however, there was no significant difference in tracer binding between the bilateral striatum, suggesting that ^18^F-EGA and ^18^F-EEA detected GFAP upregulation in rat brain sections. Furthermore, the binding of ^18^F-EGA in the presence of non-radiolabeled EGA was higher than that of ^18^F-EEA in the presence of non-radiolabeled EEA (Fig 4B and 4C), suggesting that ^18^F-EGA showed increased non-specific binding than ^18^F-EEA. Taken together, ^18^F-EEA was superior to ^18^F-EGA because ^18^F-EEA possesses a higher binding affinity for GFAP and lower non-specific binding than ^18^F-EGA.

### *In vivo* PET imaging and *ex vivo* biodistribution of ^18^F-EEA

**Fig 5:**
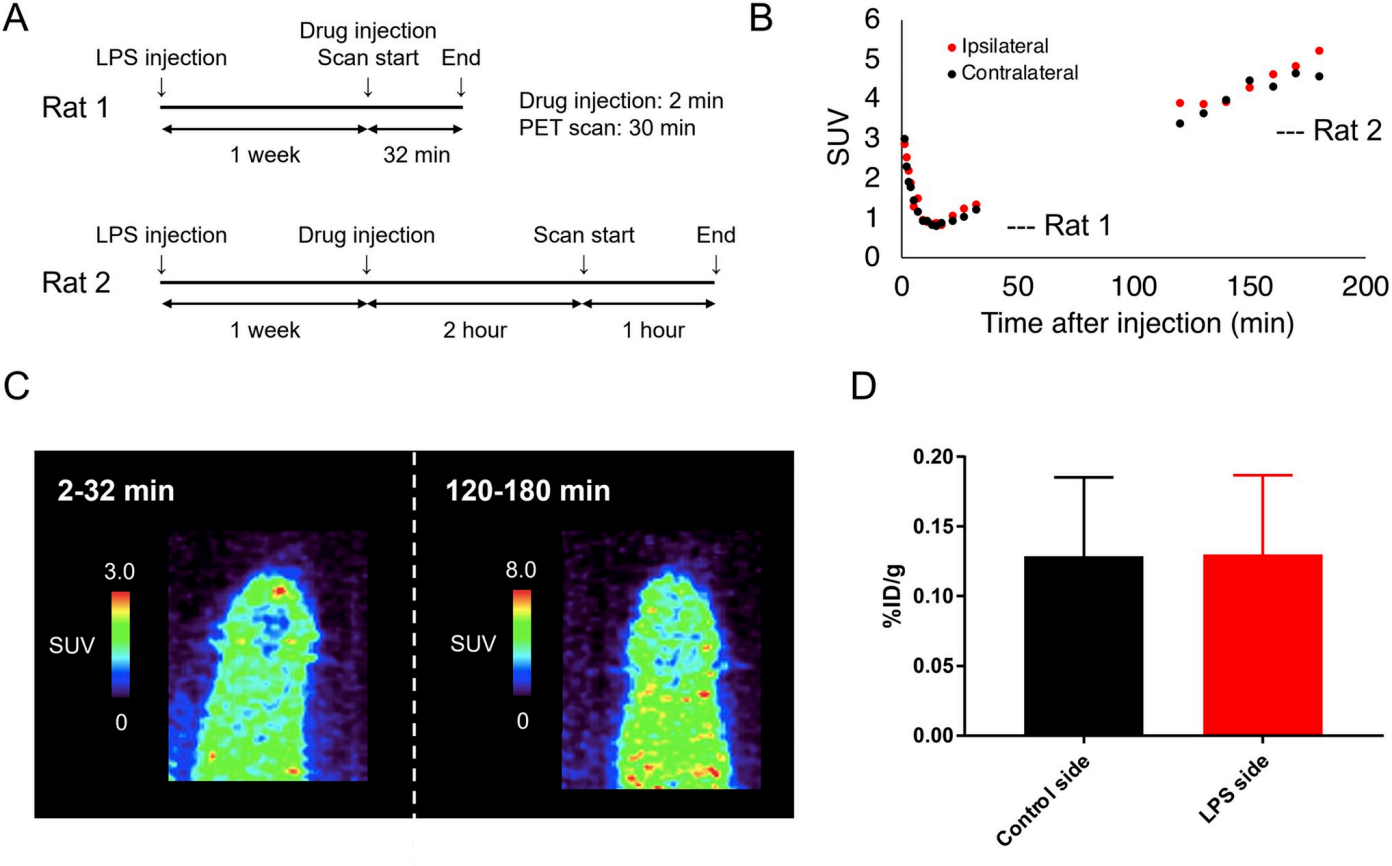
PET imaging of neuroinflammation rat models with ^18^F-EEA. (A) Experimental flow for PET scan with ^18^F-EEA. (B) Time activity curve of ^18^F-EEA in LPS-injected and contralateral side of LPS-induced rat models. (C) Accumulated PET images of LPS-induced rat models at 2–32 min and 120–180 min post intravenous administration of ^18^F-EEA. SUV = standardized uptake value. (D) Uptake of ^18^F-EEA in the left and right brains of rat models at 180 min after ^18^F-EEA intravenous injection.

To visualize neuroinflammation *in vivo*, we performed an exploratory PET and *ex vivo* biodistribution study to validate whether the *in vitro* results can be translated into living animals. We selected ^18^F-EEA for further experiments because of its higher radiochemical yield, higher binding affinity to GFAP, and lower non-specific binding to brain tissues. First, we performed a 30 min dynamic scan and 60 min static scan (from 120 min to 180 min after injection) on neuroinflammatory rats with study design described (Fig 5A). The time-activity curve of the standardized uptake value (SUV) is shown in Fig 5B. Selected brain regions for analysis were shown at S4 Fig. Brain uptake of ^18^F-EEA showed a gradual increase in rats during the 180 min scan, which is similar to the pharmacokinetics of ^18^F-AS69-ApoE, as previously described [29]. However, there were no significant differences between the LPS-injected and contralateral sides at late time points (Fig 5B and 5C). In the *ex vivo* study, we also found no significant difference in ^18^F-EEA uptake between the LPS-injected and contralateral sides at 180 min post-injection, which was consistent with the PET result (Fig 5D). S5 Fig shows ^18^F-EEA uptake in other tissues at this *ex vivo* study.

## Discussion

Neuroimaging with antibody-like affinity proteins could be a promising strategy for detecting target molecules that small molecules have difficulty recognizing such as GFAP [42,43]. However, they should overcome the problems of slow clearance and low brain permeability. Sehlin D. and co-workers reported the first demonstration of neuroimaging with radiolabeled affinity protein by fusing an anti-transferrin receptor antibody, which mediates receptor-mediated brain delivery, to an anti-soluble amyloid-β protofibril antibody derivative with ^124^I labeling [44]. The PET images took three days to acquire; however, they reduced antibody size and performed brain imaging within 14 h by fusion of two single-chain antibody fragments (scFv, 58 kDa) at their recent studies [43–47]. The fusion of small-size affinity proteins (14 kDa) and brain shuttle peptides (-3 kDa) is a possible strategy for developing smaller protein PET radiotracers that rapidly reach the brain after administration and are cleared. Moreover, our advanced CFRPS method has the unique advantage of simple protein ^18^F labeling by designing DNA that encodes the target protein sequence and applying it to the established cell-free system.

Based on previous findings of ^18^F-AS69-ApoE, we designed and engineered the GFAP-binding protein E9 by fusion with the brain shuttle peptide ApoE(159–167)_2_ [27,28]. Previously, E9 was reported to penetrate the blood-brain barrier and interact with GFAP in mice after intravenous administration because of the electrostatic effect caused by its high pI [30] (Fig 1). The pathway of protein permeation from the blood to the brain spurring by electrostatic effect is called adsorptive-mediated transcytosis [41,42] Furthermore, fusion of the brain shuttle peptide reportedly increases brain uptake via receptor-mediated transcytosis [27,28]. Hence, ApoE(159–167)_2_ fusion to E9 was expected to use both the absorptive-mediated transcytosis and receptor-mediated transcytosis pathways to send itself to the brain more effectively *in vivo*.

To evaluate the effect of the linker, we produced and compared two types of fusion proteins, EGA and EEA, in terms of bacterial expression efficiency and binding affinity against GFAP. Three molecules were successfully expressed in *E*. coli; however, the expression of EGA was lower than that of E9 and EEA due to self-aggregation during purification. This suggests that EGA may be intrinsically unstable in solution, possibly because of its higher isoelectric point. While EEA had a similar affinity for GFAP as E9, EGA had a lower binding affinity in an ELISA experiment (Fig 1C). These findings also suggest that a flexible linker may reduce the affinity of E9 for GFAP, possibly due to the overall higher pI and interaction of E9 with ApoE(159–167)_2_. The rigid linker that restricts intermolecular interactions may be beneficial in maintaining the E9 binding ability. Moreover, long rigid linkers may contribute to increased protein stability, as previously reported [37]. The radiolabeled protein tracers, ^18^F-E9, ^18^F-EGA, and ^18^F-EEA, were successfully produced using the CFRPS system (Fig 3). In CFRPS, ^18^F-E9 and ^18^F-EEA had a relatively higher radiochemical yield than ^18^F-EGA, as seen in bacterial expression, showing that radiosynthesis efficiency correlates with *E*. coli expression efficiency. This is probably because the cell-free system is made from *E*. coli extracts, and this knowledge will be useful in assuming the radiosynthesis efficiency of other proteins. At nanomolar concentrations, all radiolabeled molecules ^18^F-E9, ^18^F-EGA, and ^18^F-EEA bound GFAP to discriminate lesions of LPS-induced neuroinflammation in rat brain sections (Fig 4). Excess non-radiolabeled additives displaced the binding, indicating that these radiolabeled proteins bind to GFAP specifically. As for ^18^F-EGA and ^18^F-EEA, the binding was replaced by excess E9 to eliminate bilateral differences in the rat brain sections; however, the remaining radioactivity was higher than that of E9. This may reflect the binding of ApoE(159–167)_2_ to potential target molecules unrelated to neuroinflammation, including LRP1. In addition, the non-specific binding of ^18^F-EGA was higher than that of ^18^F-EEA (Fig 4). This result implies that the flexible linker produced non-specific binding to other targets, in addition to a reduction in the binding affinity to GFAP.

In this study, we observed no bilateral differences in the neuroinflammation rat model on PET images within 180 min of intravenous injection of ^18^F-EEA (Fig 5B and 5C). In addition, there was no significant bilateral difference in the brain 180 min after intravenous administration in *ex vivo* quantitative analysis (Fig 5D). Recently, Meier et al. reported GFAP imaging findings using ^125^I-labeled E9 nanobody derivatives in a transgenic mouse model with amyloid pathology and neuroinflammation (ArcSwe) [38]. Fusion of the anti-transferrin receptor scFv at 120 min after intravenous administration increased brain delivery by approximately two- to three-fold. *In vitro* autoradiography of scFv fusion revealed that E9 bound more strongly in ArcSwe than in the wildtype, which was consistent with our autoradiography data in the rat model (Fig 4). However, *ex vivo* autoradiography revealed no significant difference in tracer binding between ArcSwe and wildtype mice at 8 h and 48 h after intravenous administration, which was similar to our *ex vivo* findings obtained 3 h after intravenous injection of ^18^F-EEA. They suggested that this was due to the inability of protein radiotracers to reach cytosolic GFAP as opposed to extracellular amyloid plaques, which may also be true for ^18^F-EEA [38]. However, immunohistochemical analysis showed that E9 nanobody recognized astrocytic GFAP in mice 90 min after intravenous injection of 2 mg of E9 nanobody [18]. This inconsistency may be attributed to the difference in injected dose, as scFv fusion E9 and ^18^F-EEA were used at lower doses. Additionally, from PET images, it is suggested that ^18^F-EEA was widely distributed throughout the body at 180 min post-injection. Therefore, the radiotracers bound to GFAP may have been buried by background radioactivity from the blood and surrounding tissues. In this case, waiting longer to acquire PET images may result in successful visualization of neuroinflammation in rat models.

In conclusion, E9, EGA, and EEA bind to GFAP, and their radiolabeled counterparts bind to GFAP in rat brain sections to differentiate neuroinflammation. However, ^18^F-EEA could not clearly differentiate neuroinflammatory lesions in rat models *in vivo* within 180 min of intravenous administration. Therefore, to develop neuroimaging strategies that use ^18^F-labeled small affinity proteins, the type of brain shuttle peptide to be fused for better diffusion in the brain and the time range to acquire PET images should be further considered.

## Supporting information

S1 Fig Purity of strep-pCNF-RS used for cell-free protein synthesis. Purified strep-*p*CNF-RS was analyzed by SDS-PAGE. L: Ladder, P: Product.
(TIF)

S2 Fig Construction of a neuroinflammation rat model. (A) The position where LPS was injected. Red dot indicates the position of injection coordinate. Black squares and attached numbers correspond to fluorescence images at (D). Western blot analysis between LPS-injected and contralateral side of the rat brain (B) and its quantification (C). Red or green fluorescence are attributed to actin and GFAP, respectively. *: p<0.05. (D) Fluorescent images from immunohistochemical staining of a model rat brain section with anti-GFAP and anti-Iba1 antibody. Numbers correspond to the places described at (A). Scale bar: 100 μm.
(TIF)

S3 Fig Representative images from immunohistochemical staining of rat brain sections with anti-LRP1 antibody and EGA. White arrows indicate the position where the anti-LRP1 antibody and EGA colocalized. Blue indicates nuclear staining with DAPI. Scale bars: 50 μm.
(TIF)

S4 Fig Selected region of interest (ROI) for analysis of PET images used at Fig 5B. All selections of ROI were adjusted to the same volume and position using AMIDE software.
(TIF)

S5 Fig Ex vivo biodistribution of ^18^F-EEA after three hours from drug injection to rat models. (TIF)

S1 Raw images (TIF)

S2 Raw images (TIF)

S3 Raw images (TIF)

S4 Raw images (TIF)
