# Supplementary figures and images for: Evaluation of 18F labeled glial fibrillary acidic protein binding nanobody and its brain shuttle peptide fusion proteins using a neuroinflammation rat model
Source: PLoS One. 2023 Jun 14;18(6):e0287047. doi: 10.1371/journal.pone.0287047 (PMC10266665; doi:10.1371/journal.pone.0287047)

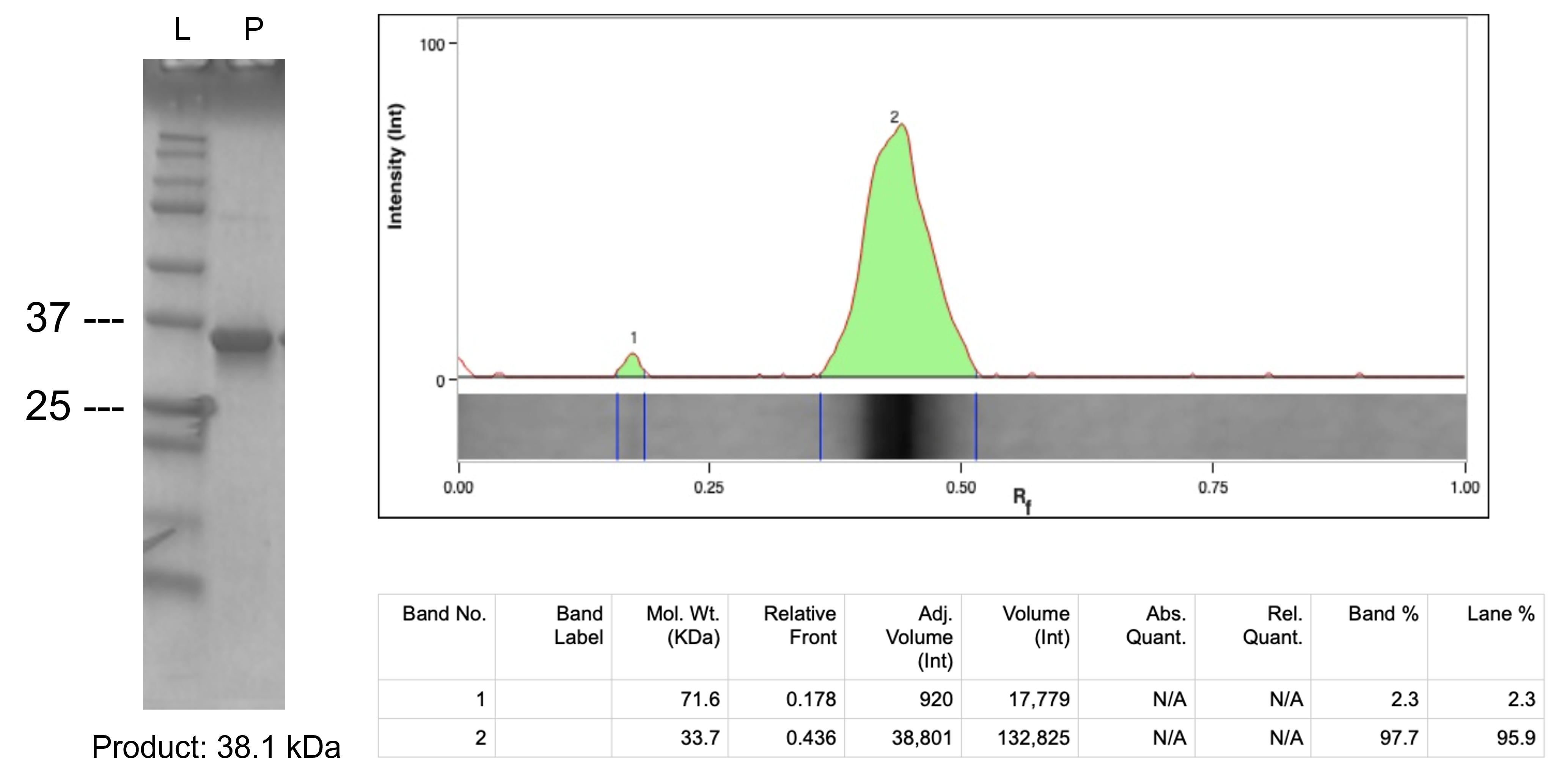

Supplement: S1 Fig — Purified strep-pCNF-RS was analyzed by SDS-PAGE. L: Ladder, P: Product. (TIF) [file pone.0287047.s001.tif]

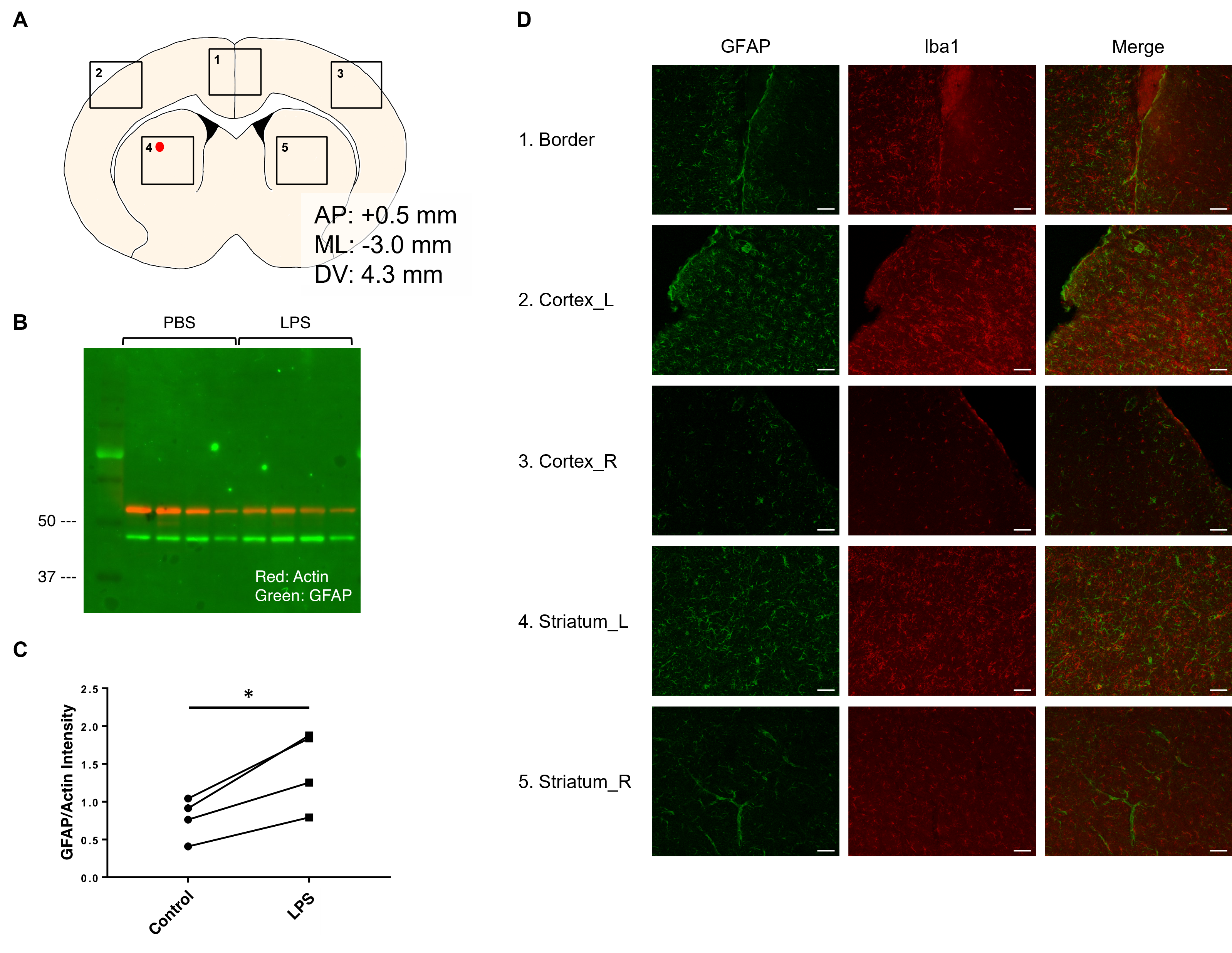

Supplement: S2 Fig — (A) The position where LPS was injected. Red dot indicates the position of injection coordinate. Black squares and attached numbers correspond to fluorescence images at (D). Western blot analysis between LPS-injected and contralateral side of the rat brain (B) and its quantification (C). Red or green fluorescence are attributed to actin and GFAP, respectively. *: p<0.05. (D) Fluorescent images from immunohistochemical staining of a model rat brain section with anti-GFAP and anti-Iba1 antibody. Numbers correspond to the places described at (A). Scale bar: 100 μm. (TIF) [file pone.0287047.s002.tif]

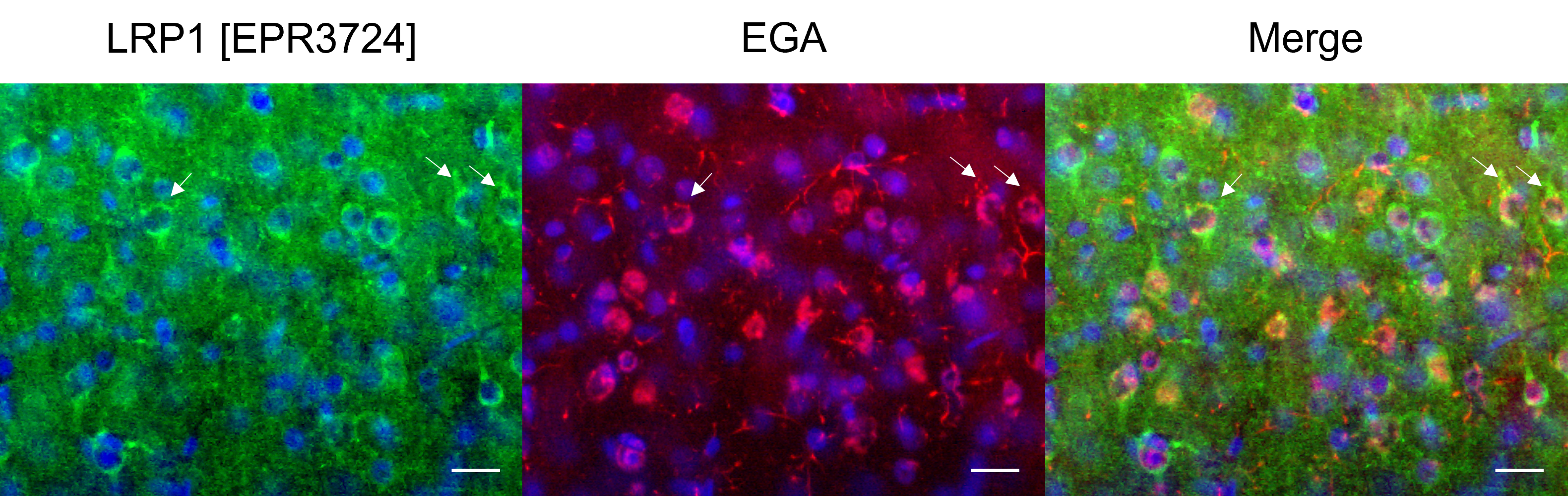

Supplement: S3 Fig — White arrows indicate the position where the anti-LRP1 antibody and EGA colocalized. Blue indicates nuclear staining with DAPI. Scale bars: 50 μm. (TIF) [file pone.0287047.s003.tif]

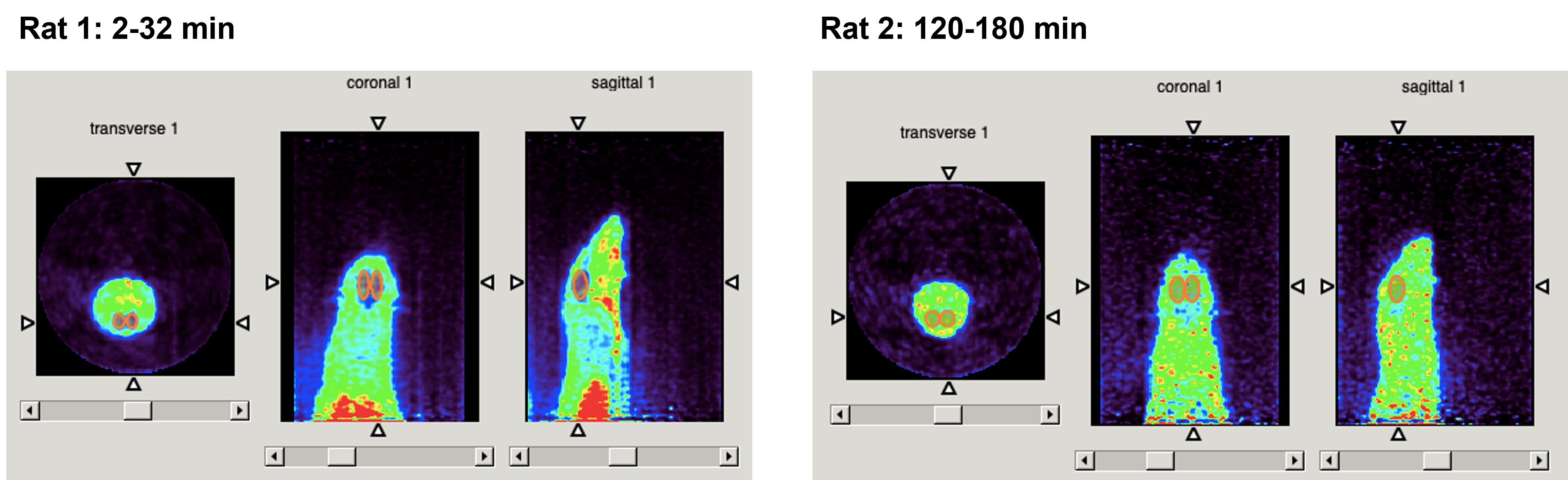

Supplement: S4 Fig — All selections of ROI were adjusted to the same volume and position using AMIDE software. (TIF) [file pone.0287047.s004.tif]

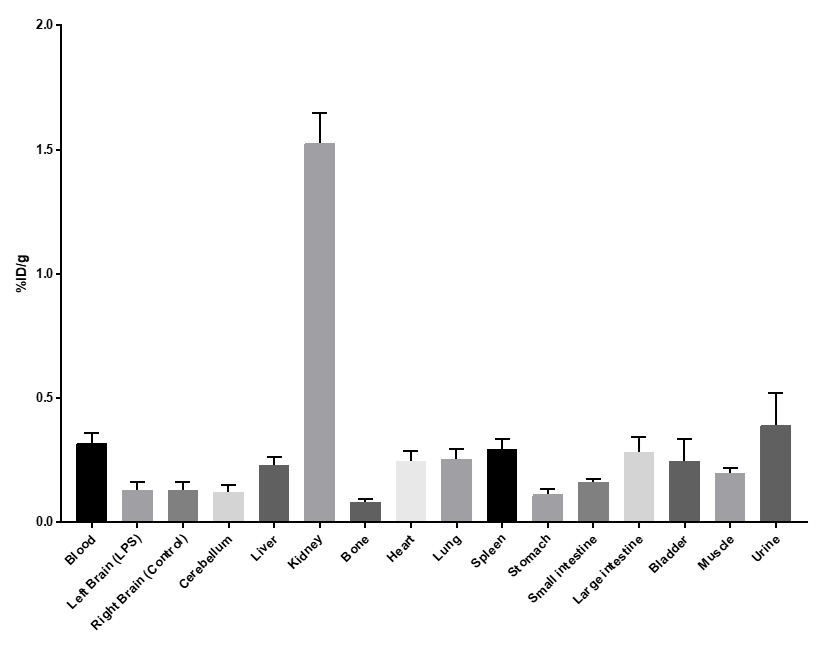

Supplement: S5 Fig — (TIF) [file pone.0287047.s005.tif]

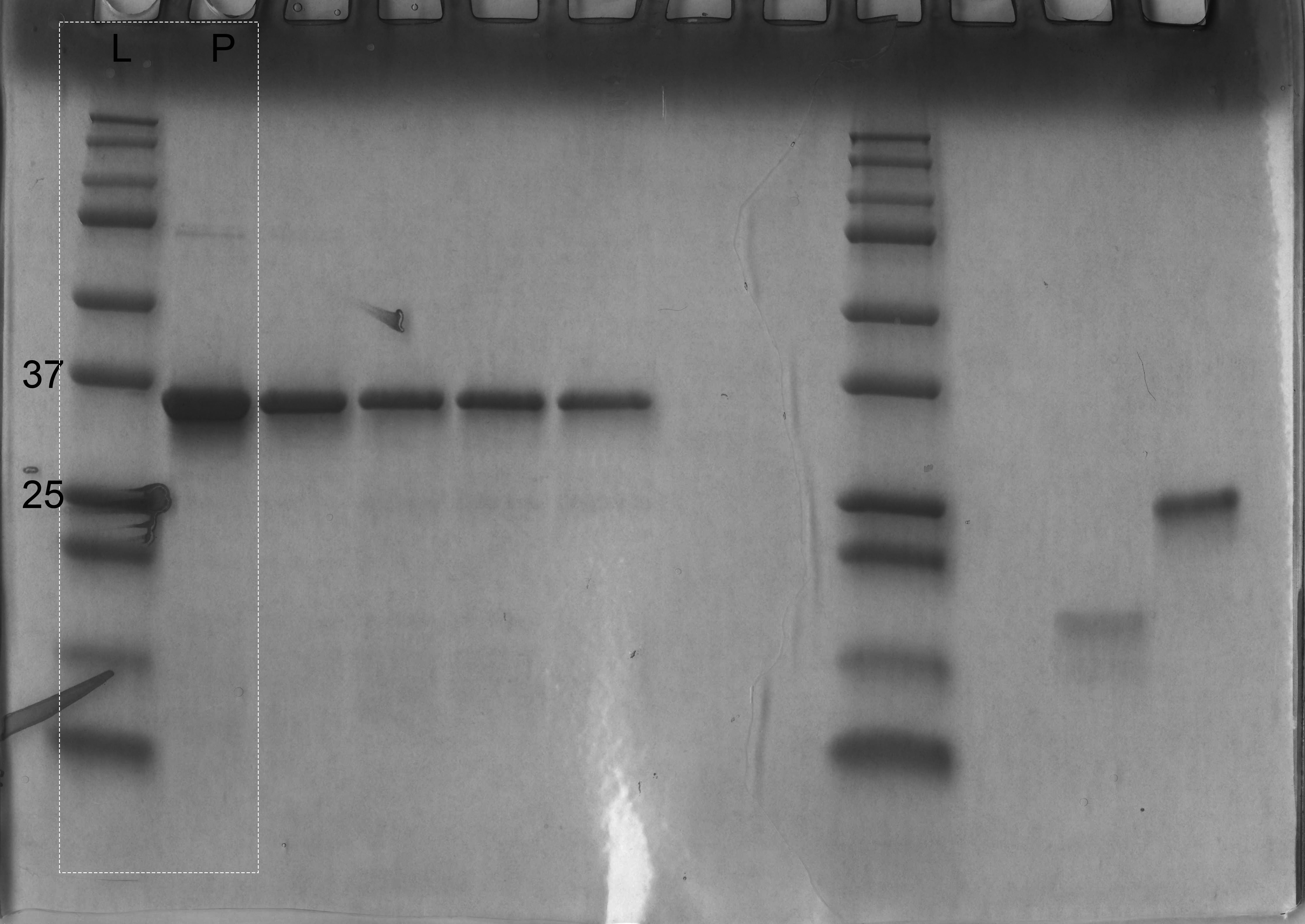

Supplement: S1 Raw images — (TIF) [file pone.0287047.s006.tif]

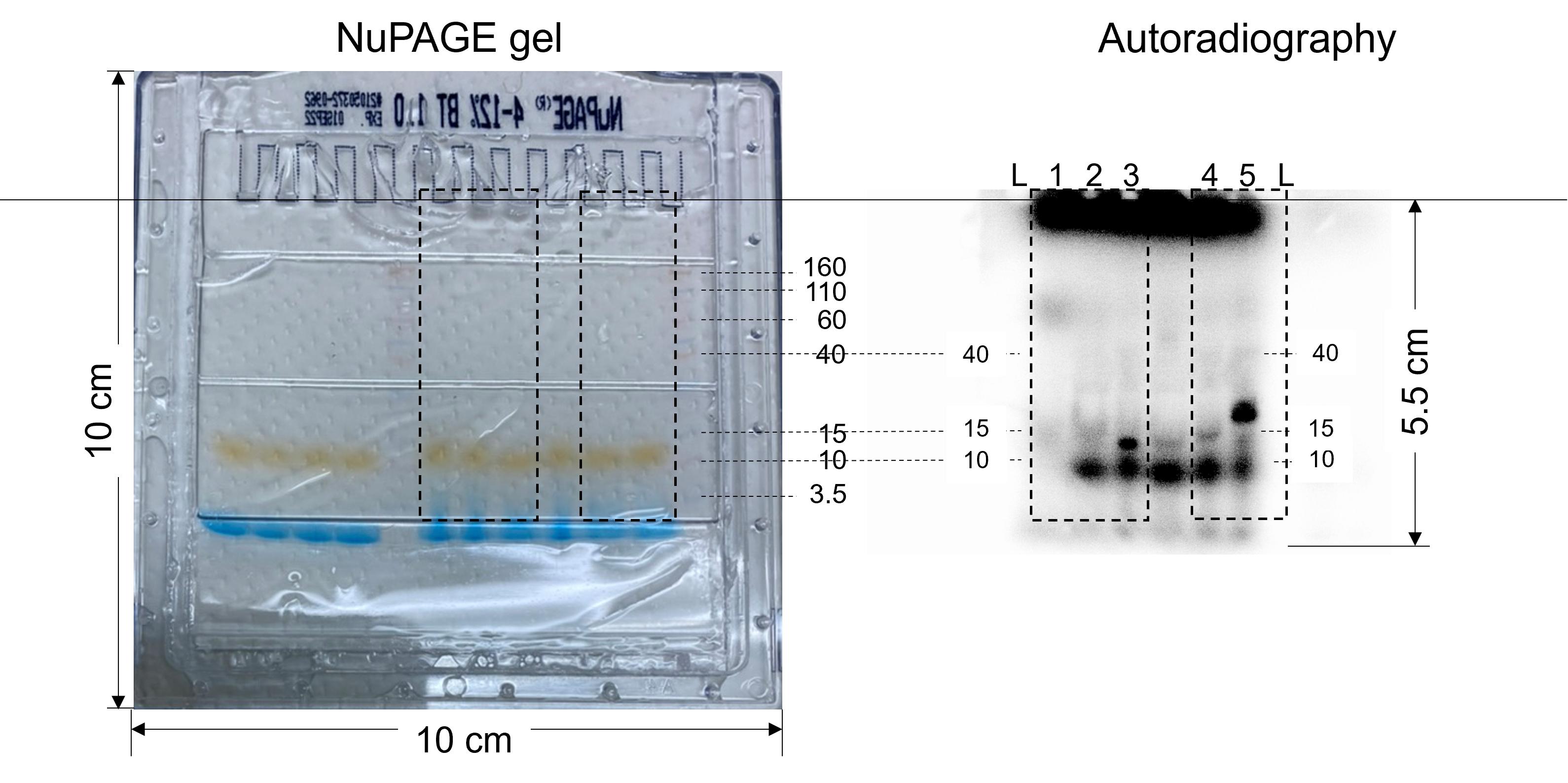

Supplement: S2 Raw images — (TIF) [file pone.0287047.s007.tif]

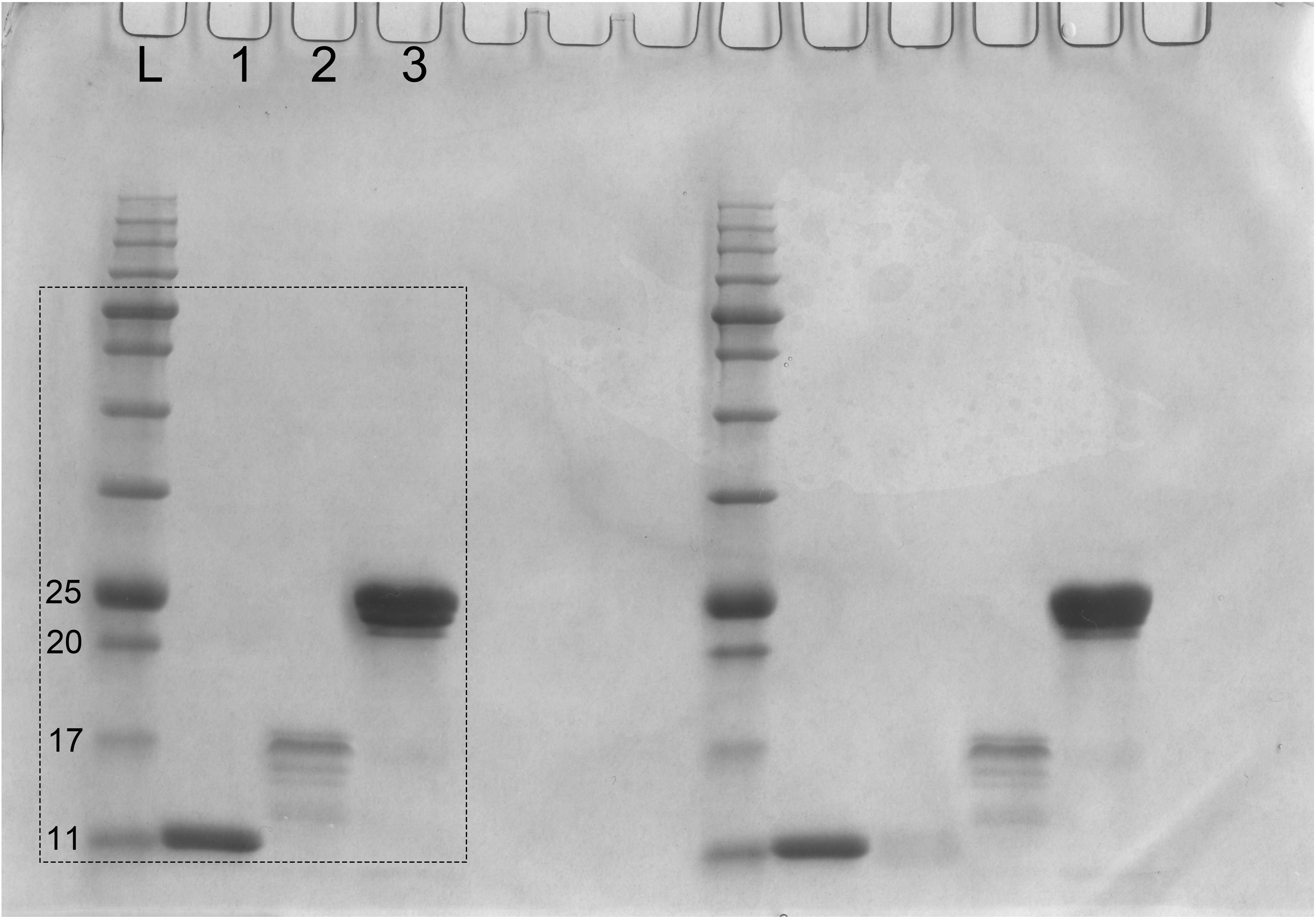

Supplement: S3 Raw images — (TIF) [file pone.0287047.s008.tif]

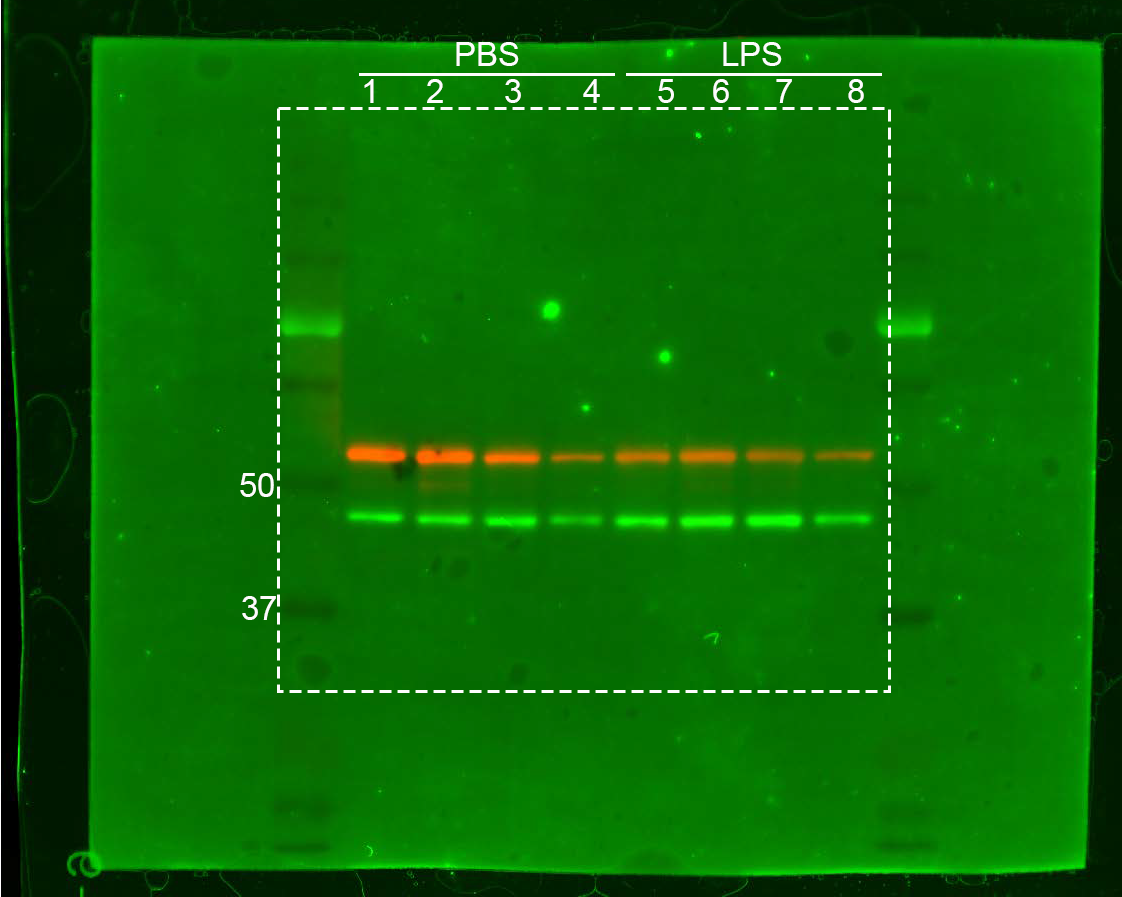

Supplement: S4 Raw images — (TIF) [file pone.0287047.s009.tif]
